# Supplementary material for: Impacts of the COVID-19 Pandemic on Food Security and Diet-Related Lifestyle Behaviors: An Analytical Study of Google Trends-Based Query Volumes
Source: Nutrients. 2020 Oct 12;12(10):3103. doi: 10.3390/nu12103103 (PMC7601866; doi:10.3390/nu12103103)
Supplement: Supplementary file 1 [file nutrients-12-03103-s001.pdf]

Supplementary Table 1.
Keyword search term in different languages

USA/UK/Singapore Philippines/ Nigeria	Spain	Italy	France	Saudi arabia	Japan	UAE	South korea	Indonesia	Malaysia	Jordan	Taiwan	Vietnam
Food bank	Banco de alimentos	Banca del cibo	Banque d'aliments	Food bank	フードバンク	Food bank	푸드뱅크	Bank makanan	Food bank	بنك الطعام	食物銀行	Nguồn cung thức phẩm
Free food	Alimento gratis	Cibo gratis	Nourriture gratuite	Free food	無料の食事	Free food	무료 식품/ 공짜 식품	Makanan gratis	Makanan percuma	Free food	食物	
Free meal	Comida gratis	Pasto gratis	Plat gratuit	Free meal	無料の食事	Free meal	무료 음식/ 공짜 음식	Makanan gratis	Makanan percuma	وجبة مجانية	餐	
Food shortage	Escasez de alimentos	Carenza di cibo	Pénurie d'aliments	نقص الغذاء	食糧不足	Food shortage	식량 부족/ 음식 부족	Kelangkaan pangan	Kekurangan makanan	نقص الغذاء	糧食短缺	Thiếu hụt thúc ăn
Food safety	Seguridad alimentaria	Sicurezza di alimentari	Sécurité alimentaire	Food safety	Food safety	Food safety	식품 안전	Keamanan pangan	Keselamatan makanan	Food safety	食品安全	An toàn thưc phẩm
Food hygiene	Higiene de los alimentos	Igiene di alimentari	Hygiène alimentaire	الصحة الغذائية	Food hygiene	Food hygiene	식품 위생	Hygiene makanan	Hygiene makanan	الصحة الغذائية	食品衛生	Vệ sinh thực phẩm
Food	Alimento	Cibo	Nourriture	طعام	Food	طعام	식품/ 음식	Pangan/makanan	Makanan	طعام	食物	Thức ăn
Meal	Comida	Pasto	Plat/ repas	وجبة	Meal	وجبة	식사	Makanan	Makanan	وجبة	餐	Bữa ăn
Breakfast	Desayuno	Colazione	Petit-dejeuner	وجبة افطار	Breakfast	وجبة افطار	아침	Sarapan	Sarapan	Breakfast	早餐	Bữa sáng
Alcohol	Alcohol	Alcool	Alcohol	الكحول	アルコール	Alcohol	술	Alkohol	Alkohol	الكحول	酒精	Cồn
Restaurant	Restaurante	Ristorante	Restaurant	مطعم	飲食店	Restaurant	식당	Restoran	Restoran	مطعم	餐廳	Nhà hàng
Delivery	Entrega	Consegna	Livraison	توصيل	配達	Delivery	배달	Pengiriman	Delivery	توصيل	交貨	Giao hàng
Food delivery	Entrega de comida	Domicilio	Livraison de nourriture	Food delivery	食品配達	Food delivery	배달 음식	Pengiriman makanan	Food delivery	Food delivery	外送	Giao hàng

Take away	Comida para llevar	Porta via	Plat à emporter	Take away	取り除く	Take away	포장	Makanan bungkus	Bungkus / bawa balik	Take away	外帶	Take away
Recipe	Receta	Ricetta	Recette	Recipe	レシピ	Recipe	레시피	Resep	Resepi	Recipe	食譜	Cách nấu
Cuisine	Cocina	Cucina	Cuisine	Cuisine	料理	Cuisine	요리	Masakan	Masakan	Cuisine	烹飪	Ẩm thực
Cake	Pastel	Torta	Gâteau	كيك	ケーキ	Cake	케이크	Cake	Kek	كيك	蛋糕	Bánh
Netflix	Netflix	Netflix	Netflix	Netflix	Netflix	Netflix	넷플릭스	Netflix	Netflix	Netflix	Netflix	Netflix
Nintendo	Nintendo	Nintendo	Nintendo	Nintendo	任天堂	Nintendo	닌텐도	Nintendo	Nintendo	Nintendo	任天堂	Nintendo
Play station	Play station	Play station	Play station	بلاي ستيشن	プレイステーション	بلاي ستيشن	플레이 스테이션	PS	PS	بلاي ستيشن	家用遊戲機	Play station
Cinema	Cine	Cinema	Cinéma	سينما	シネマ	سينما	영화관	Cinema	Pawagam	سينما	電影	Rạp chiếu phim
Hotel	Hotel	Hotel	Hotel	فندق	ホテル	Hotel	호텔	Hotel	Hotel	فندق	飯店	Khách sạn
Resort	Resort	Resort	Resort	منتجع	リゾート	Resort	리조트	Resort	Resort	Resort	度假村	Resort
Park	Parque	Parco	Parc	حديقة	パーク	Park	공원	Taman	Taman	Park	公園	Công viên
Plant	Planta	Pianta	Plante	نبات	工場	Plant	식물	Tanaman	Tanaman	نبات	植物	Thực vật
Flower	Flor	Fiori	Fleur	زهرة	花	Flower	꽃	Bunga	Bunga	زهرة	花	Hoa
Gym	Gym	Palestra	Gym	Gym	ジム	Gym	헬스장	Gym	Gym	Gym	健身房	Gym
Exercise	Ejercicio	Esercitazione	Exercise	ممارسة	運動	Exercise	운동	Olahraga	Bersenam	Exercise	運動	Thể dục
Outdoor	Afuera	Fuori	Dehors	Outdoor	アウトドア	Outdoor	야외활동	Tempat terbuka	Luar taska	Outdoor	戶外	Ngoài trời
Workout	Workout	Workout	Workout	تجريب	いい結果になる	Workout	운동	Workout	Workout	Workout	重量訓練	Thể dục
Yoga	Yoga	Yoga	Yoga	Yoga	ヨガ	Yoga	요가	Yoga	Yoga	Yoga	瑜珈	Yoga
Sunbathing	Tomar el sol	Prendere il sole	Bain de soleil	تسفع	日光浴	Sunbathing	일광욕	Berjemur	Berjemur	Sunbathing	日光浴	Tắm nắng
Cycling	Ciclismo	Ciclismo	Cyclisme	Cycling	サイクリング	Cycling	자전거 타기/사이클링	Bersepeda	Berbasikal	Cycling	騎腳踏車	Đạp xe đạp
Vitamin	Vitamina	Vitamina	Vitamine	فيتامين	ビタミン	Vitamin	비타민	Vitamin	Vitamin	فيتامين	維生素	Vitamin

Vitamin C	Vitamina C	Vitamina C	Vitamine C	فيتامين سي	ビタミンC	Vitamin C	비타민 C	Vitamin C	Vitamin C	فيتامين سي	維生素 C	Vitamin C
Vitamin D	Vitamina D	Vitamina D	Vitamine D	فيتامين د	ビタミンD	Vitamin D	비타민 D	Vitamin D	Vitamin D	فيتامين د	維生素 D	Vitamin D
Vitamin A	Vitamina A	Vitamina A	Vitamine A	فيتامين أ	ビタミンA	Vitamin A	비타민 A	Vitamin A	Vitamin A	فيتامين أ	維生素 A	Vitamin A
Vitamin E	Vitamina E	Vitamina E	Vitamine E	فيتامين هـ	ビタミンE	Vitamin E	비타민 E	Vitamin E	Vitamin E	Vitamin E	維生素 E	Vitamin E
Vitamin B	Vitamina B	Vitamina B	Vitamine B	فيتامين ب	ビタミンB	Vitamin B	비타민 B	Vitamin B	Vitamin B	فيتامين ب	維生素 B	Vitamin B
Zinc	Zinc	Zinco	Zinc	زنك	亜鉛	Zinc	아연	Zink	Zink	Zinc	鋅	Kẽm
Iron	Hierro	Ferro	Fer	حديد	鉄	Iron	철	Zat besi	Zat besi	حديد	鐵	Sắt
Selenium	Selenio	Selenio	Sélénium	السيلينيوم	セレン	Selenium	셀레늄	Selenium	Selenium	Selenium	硒	Se
Turmeric	Curcuma	Curcuma	Curcuma	الكركم	ターメリック	Turmeric	강황	Temulawak	Kunyit	الكركم	薑黃	Nghệ
Garlic	Ajo	Aglio	Ail	ثوم	ニンニク	Garlic	마늘	Bawang putih	Bawang putih	ثوم	蒜頭	Tỏi
Ginger	Jenjibre	Zenzero	Gingembre	زنجبيل	ショウガ	Ginger	생강	Jahe	Halia	زنجبيل	薑	Gừng
Onion	Cebolla	Cipolla	Oignon	بصل	玉ねぎ	Onion	양파	Bawang bombay	Bawang merah	بصل	洋蔥	Hành
Herbal	Hierba	Erbacea	Plantes medicinales	Herb	ハーブ	Herb	약초/ 허브	Herbal	Herba	أعشاب	草本植物	Thảo mộc
Omega 3	Omega 3	Omega 3	Oméga 3	أوميغا 3	オメガ3	Omega 3	오메가 3	Omega 3	Omega 3	Omega 3	Omega-3 脂肪酸	Omega 3
Cookies	Galletas	Biscotto	Biscuits	بسكويت	クッキー	Cookies	과자	Cookies	Biskut	بسكويت	餅乾	Bánh cookie
Bread	Pan	Pane	Pain	خبز	パン	Bread	빵	Roti	Roti	Bread	麵包	Bánh mì
Pizza	Pizza	Pizza	Pizza	بيتزا	ピザ	Pizza	피자	Pizza	Pizza	بيتزا	披薩	Pizza
Chicken	Pollo	Pollo	Poulet	دجاج	チキン	Chicken	치킨	Ayam	Ayam	دجاج	雞肉	Thịt gà
Herbal remedy	Fitoterapia	Erbe	Phytotérapie	علاج عشبي	薬草剤	Herbal remedy	한방 치료/ 한방약	Jamu	Ubat herba	علاج عشبي	草藥	Dược thảo
Vegetables	Legumbres	Verdure	Légumes	خضروات	野菜	Vegetables	채소	Sayuran	Sayur-sayuran	خضروات	蔬菜	Rau xanh
Immunity	Inmunologia	Immunita'	Immunologie	مناعة	免疫	Immunity	면역	Daya tahan tubuh	Immuniti	Vegetables	免疫	Miễn dịch

Beer	Cerveza	Birra	Bière	بيرة	ビール	Beer	맥주	Bir	Bir	Beer	啤酒	Bia
Take out	Eliminar	Take out	Sortir	Take out	取り出す	Take out	포장	Bungkus	Bungkus / bawa balik	تخلص من	外帶	Take away
Curcumin	Cúrcumin	Curcumina	Curcumine	الكركم	クルクミン	الكركم	커큐민	Kunyit	Kunyit	الكركم	薑黃素	Curcumin
Weigh loss	Perdida de peso	Perdita di peso	Perte du poids	Weigh loss	減量	فقدان الوزن	체중 감량/ 체중 감소	Penurunan berat badan	Penurunan/ kejatuhan berat badan	فقدان الوزن	減肥	Giảm cân
Plant-based diet	Dieta a base de plantas	Dieta a base vegetale	Régime à base de plantes	Plant-based diet		النظام الغذائي النباتي		Diet vegan	Plant-based diet	النظام الغذائي النباتي		Chế độ ăn thuần thực vật
Ketogenic diet		Dieta chetogenica	Régime cétogène	Ketogenic diet				Diet keto	Ketogenic diet			Giảm cân keto
Coffe	Café	Cafe'	Café	قهوة	コーヒー	Coffee	커피	Kopi	Kopi	قهوة	咖啡	
Fitness	Fitness	Fitness	Fitness	Fitness	フィットネス	Fitness	피트니스	Fitness	Fitness	Fitness	Weigh loss	
Aerobics	Aeróbico	'Aerobica	Aérobique	التمارين الرياضية	エアロビクス	Aerobics	에어로빅	Aerobik	Aerobik	التمارين الرياضية	Coffe	Aerobic
Meditation	Meditacion	Meditazione	Méditation	تأمل	瞑想	Meditation	명상	Meditasi	Meditasi	تأمل		Thiền
Relaxation	Relajaxion	Rilassamento	Relaxation	استرخاء	リラクゼーション	Relaxation	휴식	Relaksasi	Relak	استرخاء		Thư giãn

Supplementary Table 2.
Checklist for Documentation of Google Trends research. Modified from Nuti et al.

Section/Topic	Checklist item
**Search Variables**	
Access Date	31 April 2020
Time Period	From 1 June 2019 to 27 April 2020
Query Category	Search term
Region	Worldwide and regional (21 countries across Asia (Taiwan, China, Hong Kong, Singapore, Indonesia, Malaysia, Philippine, Vietnam, South Korea, Japan), Europe (Italy, France, Spain, UK), Middle East (Egypt, Dubai, Jordan, Saudi Arabia), North America (USA) and Africa (Nigeria, Kenya)
Countries with Low Search Volume	Excluded
**Search Input**	
Full search input	(1) Coronvirus: "Coronavirus", "Covid-19", "Covid 19", "Covid", "SARS-CoV2"<br>(2) Food security: "food bank", "free food", "free meal", "food shortage".<br>(3) Dietary behaviour: "restaurant", "delivery", food delivery" "take away"<br>(4) Lifestyle behaviour: "Netflix", "Nintendo", "recipe", "cuisine", "cake","cinema", "hotel", "resort", "park". Terms like "gym", "exercise", "outdoor", "plant", "workout", "yoga", "sunbathing", "cycling", "fitness", "aerobics", "plant",<br>(5) Immune-related nutrients and herbs: "vitamin", "vitamin A", "vitamin B", "vitamin C", "vitamin D", "vitamin E", "zinc", "omega 3", "iron", "selenium", "herb", "turmeric", "garlic", "ginger", "onion"
Quotaion Marks	If there was one word in search term, those words were queried with quaotation marks
**Rationale for Search Strategy**	
For Search Input	The searched terms were related to change in diet and lifestyle behaviour during coronvirus pandemic
For Setting Chosen	Web search was choosen
**Hypothetical example**	
On 31 April 2020, we queried Google Trends and downloaded the data for the above search input. We searched the wordwide and regional data from 1 June 2019 to 27 April 2020 with no restriction on the query category. We search those terms based on the hypothesis that our diet and lifestyle behavour may change during the Covid-19 pandemic may affect. We chose 1 June 2019 as the start date to capture baseline interest in the half year before the Covid-19 outbreak occurded.	

S.V. Nuti, B. Wayda, I. Ranasinghe, S. Wang, R.P. Dreyer, S.I. Chen, K. Murugiah, The Use of Google Trends in Health Care Research: A Systematic Review, PLoS ONE. 9 (2014) e109583. doi:10.1371/journal.pone.0109583

Suplementary Table 3.
Top five countries in search keyword related to dietary and lifestyle behavior.

**Keyword**	**Top 1**	**Top 2**	**Top 3**	**Top 4**	**Top 5**
Restaurant	France	Switzerland	Germany	Nederlands	Spain
Delivery	Brazil	Philippines	Russia	UK	Sri Lanka
Food Delivery	Sri Lanka	Singapore	USA	Malaysia	Philippines
Netflix	Turkey	Brazil	Mexico	Italy	Spain
Recipe	New Zealand	South Africa	United States	Australia	Philippines
Cake	Indonesia	India	Malaysia	Singapore	UK
Nintendo	Japan	Germany	Spain	Netherlands	Mexico
Cuisine	France	Belgium	Switzerland	Canada	Spain
Hotel	Columbia	Spain	Mexico	Argentina	Austria
Park	United States	South Africa	Australia	New Zealand	Canada
Cinema	Brazil	Egypt	Italy	Romania	France
Resort	Russia	Thailand	Philippines	Bangladesh	Vietnam
Outdoor	Germany	Turkey	Switzerland	Brazil	Thailand
Exercise	Pakistan	Hongkong	Nigeria	India	Philippines
Gym	Mexico	Argentina	Spain	France	Sweden
Plant	Nigeria	Netherlands	Philippines	Belgium	Kenya
